# Antibiofilm Effects of Nanoparticles and Visible Light Illumination Against *Listeria monocytogenes*

**DOI:** 10.3389/fmicb.2021.710954

**Published:** 2021-07-13

**Authors:** Sanna Puranen, Kati Riekkinen, Jenni Korhonen

**Affiliations:** Institute of Public Health and Clinical Nutrition, University of Eastern Finland, Kuopio, Finland

**Keywords:** *Listeria monocytogenes*, biofilm, nanoparticle, blue light, food safety

## Abstract

*Listeria monocytogenes* bacteria pose a particular risk to the food industry as the species is known to form biofilm and to survive in a wide range of challenging environmental conditions. *L. monocytogenes* can cause listeriosis, a serious food-borne disease, and effective and safe antibiofilm materials and sanitary methods for food processing environments are intensively sought. A variety of nanoparticle materials have been recognized as safe to use in food environments, which allows the application of nanomaterials also for food safety purposes. Nanoparticles together with light illumination generate reactive oxygen species which inactivate bacteria by breaking down cell membranes, proteins, and DNA. The main objective of this study was to evaluate the efficacy of nanomaterials and blue light illumination for *L. monocytogenes* ATCC 7644 biofilm inactivation. Biofilm was allowed to form for 72 h on nanocoated stainless steel and aluminum plates, after which the plates were illuminated. Non-coated control plates were used to evaluate the antibiofilm efficacy of nanocoating. Plate count method was used to evaluate bacteria counts after illumination. Nanocoating did not affect initial biofilm formation compared to the control plates. Biofilm was significantly (*p* < 0.05) reduced on stainless steel, aluminum, and TiO_2_-coated aluminum plates after 72-h illumination by 1.9, 3.2, and 5.9 log, respectively. Nanocoating with visible light illumination could be an effective and safe method for enhancing food safety in food processing facilities to control biofilm formation. Evidence of antibiofilm properties of nanomaterials together with visible light illumination is limited; hence, future studies with variable light intensities and nanomaterials are needed.

## Introduction

*Listeria monocytogenes* bacteria are a serious risk for the food industry as they form biofilm on a variety of surfaces and can survive in a range of challenging environmental conditions (McKenzie et al., 2013; Li et al., 2018; Lee et al., 2019). The pathogen can grow at refrigeration temperatures and survive in a wide pH range and at high osmotic stress (Bucur et al., 2018), making it highly challenging to inactivate. Biofilm control is an important food safety and public health concern as *L. monocytogenes* can cause listeriosis, a serious food-borne disease, especially for pregnant women and the elderly (Farber and Peterkin, 1991). The European Center for Disease Prevention and Control (ECDC) reports that in the EU/EEA, 30 countries reported 2,502 listeriosis cases in 2017 and cases show an increasing trend (ECDC, 2020), possibly due to the increasing size of the elderly population (European Food Safety Authority, 2018).

Biofilm allows bacteria to adhere to surfaces. It develops by irreversible attachment of bacteria, maturation, and dispersion, and is one of the major means by which bacteria spread and cause contamination (Petrova and Sauer, 2016). Biofilm can be formed on any surface, but by using nanomaterial coatings, individual bacterial cells attached to the surface can be destroyed before they start to form biofilm and thus reduce the risk of contamination and possible infections (Aponiene et al., 2017). Nanomaterials are 1–100 nm size particles, such as titanium or zinc oxides that can produce free radicals during illumination leading to the death of bacteria cells by damaging cellular components such as membrane, proteins, and DNA (Khezerlou et al., 2018). The general properties of nanomaterials, such as hardness, hydrophobicity, large surface area, and optical characteristics (Khan et al., 2019; Chan et al., 2021), make them excellent surface coatings, but they can also provide surfaces with highly beneficial self-cleaning and antimicrobial properties. TiO_2_-coatings are used, for example, in public areas to ensure surface hygiene and to facilitate sanitation (Nanoksi, 2021).

Visible light illumination together with nanoparticle coating has been shown to be effective in controlling biofilm (Aponiene et al., 2017), although studies using visible light illumination and nanoparticles against *L. monocytogenes* biofilm are limited. A recent study by Aponiene et al. (2017) found that *L. monocytogenes* biofilm was reduced by 4 log from a plastic surface after zinc oxide nanoparticle coating and 405 nm light illumination (34.6 J/cm^2^). Another study investigating biofilm resistance has shown that after 400 nm illumination together with zinc oxide nanoparticle coating, planktonic listeria cells underwent 7-log reduction after exposure of 17.3 J/cm^2^ (Kairyte et al., 2013). In addition, titanium dioxide (TiO_2_) nanoparticles have been found to enhance the antibiofilm properties of UV light (Chorianopoulos et al., 2011). Visible light and nanomaterials could be used in the food industry to ensure, among other cleaning measures, the hygiene of surfaces.

Surface contamination is the most likely route for listeria contamination of food products. A study evaluating listeria in fish factories found that up to 7.2% of process surfaces were contaminated, even though the surfaces were cleaned before taking the samples (Miettinen et al., 2001). In another study, listeria was found in a pork processing plant, especially the brining machine, with 35% of the finished products found positive for *L. monocytogenes* (Berzins et al., 2010). In the food industry, a variety of methods, such as sanitizers, enzymes, heat, and non-thermal treatments such as UV light, have been used to control biofilm formation (Galié et al., 2018), and new methods are constantly being developed and studied (Gray et al., 2018). The main objective of this study was to evaluate the photocatalytic activity of TiO_2_-nanomaterial-coated surfaces against *L. monocytogenes* ATCC 7644 biofilm.

## Materials and Methods

### Bacterial Cultures and Growth Conditions

Bacterial culture of *L. monocytogenes* ATCC 7644 was inoculated into 10 ml tryptone soy broth medium (TSB, Lab M) and grown overnight (16 h, +37°C). The bacterial culture was sedimented by centrifugation (2,900 × g, 5 min) after which the pellet was resuspended in 0.9% NaCl-peptone solution. Bacterial optical density was set to 0.1–0.15 (*λ* = 625 nm) using spectrophotometry to achieve a bacteria culture of approximately 10^8^ cfu/ml. This suspension was further a 100-fold diluted in TSB. Aluminum and stainless steel plates (8 cm × 2 cm) with or without nanocoating were placed into petri dishes and submerged in TSB with *L. monocytogenes* at the desired dilution. Samples were incubated at +37°C for 72 h to allow biofilm formation. The incubation plates were then washed three times with 0.9% NaCl solution in an orbital shaker for 3 × 1 min at 200 rpm (Heidolph Unimax 2010) to remove unattached bacteria.

### Materials

Aluminum and stainless steel plates with and without TiO_2_ nanocoating were used as samples. Before each experiment, the plates were disinfected and washed with water and detergent, rinsed with distilled water, and wiped with 70% ethanol. The plates were then sterilized at +160°C for 180 min. Triplicates of the samples were used in each experiment.

### LED Light Source and Illumination Conditions

A LED light source emitting 405 nm light was used for biofilm inactivation. The light intensity was measured using a thermal power sensor S314C (Thorlabs). Light intensity was 1.0–1.4 mW/cm^2^ and power 20 W. The light source was attached to the wall of the refrigerator (Porkka Finland Oy) vertically, so the light hit the samples horizontally. Distance from light to samples was 30 cm. Temperature was set at +5 ± 2°C. The light dose was calculated as E = Pt, where E is the energy density (J/cm^2^), P is the power density (W/cm^2^), and t is time (seconds).

### Detachment of Biofilm

After light exposure, the samples were transferred into sterile 50 ml falcon tubes and 50 ml of 0.9% NaCl-peptone was added. The samples were sonicated for 3 min at 35 kHz at 120% power (Elma, Transsonic Digital S) to detach the listeria cells from the plates (Bjerkan et al., 2009; Li et al., 2018). The sample tubes were vortexed (30 s, 5000 rpm), and the desired dilutions plated onto tryptone soy agar (TSA, VWR) plates. The TSA plates were incubated for 48–72 h at +37°C before colony counting.

### Statistics

IBM SPSS Statistics 27 software was used for statistical analysis. Data were not normally distributed, so a nonparametric test with Bonferroni correction was performed. Values of *p* lower than 0.05 were considered statistically significant.

## Results

Effects of TiO_2_ nanocoating and visible light illumination (405 nm, 0–311 J/cm^2^) on *L. monocytogenes* biofilm are presented in Tables 1 and 2, which show the cumulative light dose and the results from the dark controls. After primary biofilm attachment of approximately 6 log (CFU/ml), listeria cells were attached to every sample irrespective of the material or TiO_2_ nanocoating (Tables 1 and 2).

**Table 1:** Number of *Listeria monocytogenes* on stainless steel plates after 0–72-h 405 nm light illumination at +5 ± 2°C.

Illumination 405 nm (h)	Light dose (J/cm^2^)	Stainless steel, light	Stainless steel, dark	TiO_2_-coated stainless steel, light	TiO_2_-coated stainless steel, dark
0	0	5.8 ± 0.119^a^	5.8 ± 0.119	5.9 ± 0.142	5.9 ± 0.142
2	8.6	4.2 ± 0.111^c^	5.1 ± 0.554	4.8 ± 0.286	5.6 ± 0.064
12	51.8	5.4 ± 0.251	5.2 ± 0.113	4.9 ± 0.147	5.3 ± 0.312^b^
24	13.7	4.4 ± 0.070^d^	5.4 ± 0.207	4.7 ± 0.043	5.7 ± 0.170
48	207.4	4.1 ± 0.150	5.7 ± 0.182	4.2 ± 0.582^e^	6.0 ± 0.026
72	311.0	3.9 ± 0.300	6.0 ± 0.187	3.8 ± 0.965	6.1 ± 0.047

Results are represented as logarithmic colony forming units in ml, with ± standard deviation of three replicates. Different letters indicate significant (*p* < 0.05) difference between samples.

a Significant difference from stainless steel, 72 h.

b Significant difference from TiO_2_-coated stainless steel, dark 72 h.

c Significant difference from TiO_2_-coated stainless steel, dark 2 h.

d Significant difference from TiO_2_-coated stainless steel, dark 24 h.

e Significant difference from TiO_2_-coated stainless steel, dark 48 h.

**Table 2:** Number of *L. monocytogenes* on aluminum plates after 0–72-h 405 nm light illumination at +5 ± 2°C.

Illumination 405 nm (h)	Light dose (J/cm^2^)	Aluminum, light	Aluminum, dark	TiO_2_-coated aluminum, light	TiO_2_-coated aluminum, dark
0	0	6.1 ± 0.374^a^	6.1 ± 0.374^b^	5.9 ± 0.107^c^	5.9 ± 0.107^d^
2	8.6	4.6 ± 0.163	4.4 ± 0.399	4.8 ± 0.370	5.1 ± 0.109
12	51.8	5.1 ± 0.448	5.2 ± 0.272	5.0 ± 0.335	5.3 ± 0.168
24	13.7	4.3 ± 0.363	4.8 ± 0.414	3.7 ± 0.369	4.8 ± 0.178
48	207.4	3.1 ± 0.250	4.7 ± 0.391	1.7 ± 0.310	4.9 ± 0.374
72	311.0	2.9 ± 0.328	4.7 ± 0.226^e^	0.0 ± 0.000	1.8 ± 0.285

Results are represented as logarithmic colony forming units in ml, with ± standard deviation of three replicates. Different letters indicate significant (*p* < 0.05) difference between samples.

a Significant difference from aluminum, 72 h.

b Significant difference from aluminum, dark 2 h.

c Significant difference from TiO_2_-coated aluminum, 72 h (*p* < 0.01).

d Significant difference from TiO_2_-coated aluminum, dark 72 h (*p* < 0.01).

e Significant difference from TiO_2_-coated aluminum, 72 h.

### Stainless Steel

On both illuminated stainless steel plates, biofilm was reduced approximately 2 log after 72 h compared to initial attachment before light illumination. There were no significant differences between stainless steel and TiO_2_-coated stainless steel at any time point, but listeria biofilm was significantly (*p* < 0.05) reduced from stainless steel after 72-h illumination. No antibiofilm effects were observed in the dark controls (Table 1).

### Aluminum

Listeria biofilm was significantly (*p* < 0.01, p < 0.05) reduced from illuminated TiO_2_-coated aluminum and non-coated aluminum plates by 5.9 and 3.2 log, respectively. Also, in the dark control of TiO_2_-coated aluminum, the number of listeria cells was significantly (p < 0.01) reduced after 72 h (Table 2).

## Discussion

Antibiofilm effect of TiO_2_-nanomaterial coating together with visible light illumination against *L. monocytogenes* was studied on stainless steel and aluminum surfaces. 405 nm visible light illumination was used to activate the antimicrobial properties of the nanomaterial, i.e., reactive oxygen species production, which could be one of the mechanisms behind the antimicrobial results (Khezerlou et al., 2018).

### Discussion of Results

#### Stainless Steel

Nanomaterial coatings have been reported to possess antibacterial effects in previous studies (Aponiene et al., 2017; Krumdieck et al., 2019). However, in this study, the nanomaterial did not have an additional antibacterial effect against *L. monocytogenes* compared to solely visible light illumination on stainless steel. With or without nanomaterial, the number of listeria cells decreased similarly after 72-h illumination (Table 1). Reduction of listeria from non-coated stainless steel plate is similar to the Li et al. (2018) study, where listeria biofilm was significantly reduced from stainless steel after 405 nm illumination. Although TiO_2_ has been found to have antibacterial properties, a study by Chung et al. (2009) found that the growth of bacteria on stainless steel with TiO_2_ thin film under visible light illumination did not differ from the control stainless steel surface without TiO_2_. Those observations are similar to the findings of the present study, in which no antibiofilm differences were observed between TiO_2_-stainless steel and the control. On stainless steel, listeria increased in the dark control at chilled temperatures, which shows the persistence of listeria, but also indicates that nanomaterial requires light to activate reactive oxygen species formation (Chorianopoulos et al., 2011; Shim et al., 2016; Aponiene et al., 2017).

The ineffectiveness of the nanomaterial on stainless steel could be partly explained by the horizontal light angle. Also, the finishing of surface material could be a challenge when applying nanomaterial. For example, the hydrophobicity of stainless steel may also explain the similarity of the results between TiO_2_-coated and non-coated stainless steel. Stainless steel is a versatile surface material, but multiple findings indicate that it may not be the definitive choice when seeking self-cleaning properties for food contact surfaces.

#### Aluminum

TiO_2_ nanomaterial coating had an additional antibiofilm effect compared to solely illumination of the aluminum surface. Approximately 3-log reduction was found between TiO_2_-coated aluminum and aluminum samples after 72-h illumination (Table 2). The number of listeria cells decreased more from aluminum than stainless steel surfaces, which could be explained by aluminum oxide (Al_2_O_3_) formation on the surface of aluminum. Al_2_O_3_ has been found to inhibit bacterial growth (Doskocz et al., 2017; Sikora et al., 2018), although in this study the antibacterial effect of Al_2_O_3_ is not visible at time point 0 h, since the initial attachment of listeria cells is similar between stainless steel and aluminum (Tables 1 and 2). The combined effect of Al_2_O_3_ and nanomaterial could be the reason for the complete inactivation of listeria from the TiO_2_-coated aluminum plate after 72-h illumination. Interestingly, on the dark control plate of TiO_2_-coated aluminum, the number of listeria cells decreased more efficiently than on the dark non-coated aluminum plate (Table 2). The illumination of surfaces prior to carrying out existing sanitizing practices could significantly accelerate the effectiveness of these methods and, in general, increase the microbiological safety of food processing environments.

### Limitations and Future Research Aspects

The limitations in this study relate to the intensity and orientation of the illumination used. The light falls on the samples horizontally, which lowers the light intensity. For surface disinfection, illumination should be perpendicular to the surface to attain even sanitation across the surface so that possible uneven areas do not limit the effectiveness of the light and the nanomaterial. The research frame of our study was limited by the actual circumstances that are possible to achieve with the illumination method.

It is well known that the intensity of the light exposure affects to the results obtained. Therefore, the circumstances in the research frame were kept the same, including the distance and orientation of the LED to the plates. This, however, limits the number of strains and surface materials tested simultaneously. Well-established *L. monocytogenes* ATCC 7644 strain was chosen to enable international comparison of results between studies. Research by Manso et al. (2020) showed that different *Listeria monocytogenes* isolates differ in susceptibility for environmental stress; therefore, other listeria strains should be further investigated in the future to accomplish knowledge how different strains react to nanomaterials and illumination.

More studies are needed to gain deeper knowledge of the antibiofilm properties of nanoparticles on different materials and to identify the most optimal surfaces for different applications. In addition, for safety and user-friendly purposes, further optimization studies with different light exposures and nanomaterials are needed in order to achieve as high as possible antimicrobial efficacy at as low as possible light intensity. Furthermore, the durability of different nanomaterials against chemical and mechanical cleaning needs to be investigated and improved.

## Conclusion

*Listeria monocytogenes* poses a threat to the food industry by forming biofilm that is difficult to inactivate; thus, new sanitation methods for biofilm control are being intensively investigated. Our study showed that visible light and TiO_2_ nanocoating together are effective on aluminum surface to inactivate *L. monocytogenes* at chilled temperatures. On stainless steel, TiO_2_ nanomaterial did not enhance the antibacterial effect of the surface compared to the non-coated surface. However, the amount of listeria was remarkably reduced by the effect of visible light. These results indicate that nanomaterial coating could increase the hygiene of certain surfaces compared to light exposure alone. This concept, known as the Hurdle effect, highlights the need to use multiple methods simultaneously to control or even eliminate the threat of *L. monocytogenes* in food environments.

## Data Availability Statement

The original contributions presented in the study are included in the article/supplementary material, further inquiries can be directed to the corresponding author.

## Author Contributions

SP, KR, and JK contributed to the design and implementation of the research, to the analysis of the results, and to the writing of the manuscript. All authors contributed to the article and approved the submitted version.

### Conflict of Interest

The authors declare that the research was conducted in the absence of any commercial or financial relationships that could be construed as a potential conflict of interest.
